# Transcriptomics analysis of the flowering regulatory genes involved in the herbicide resistance of Asia minor bluegrass (*Polypogon fugax*)

**DOI:** 10.1186/s12864-017-4324-z

**Published:** 2017-12-06

**Authors:** Fengyan Zhou, Yong Zhang, Wei Tang, Mei Wang, Tongchun Gao

**Affiliations:** 1 Institute of Plant Protection and Agro-Products Safety, Anhui Academy of Agricultural Sciences, Hefei, 230001 China; 2 State Key Laboratory of Rice Biology, China National Rice Research Institute, Hangzhou, 311400 China

**Keywords:** RNA sequencing, Transcriptomics, Herbicide resistance, *Polypogon fugax*, Clodinafop-propargyl, Flowering

## Abstract

**Background:**

Asia minor bluegrass (*Polypogon fugax, P. fugax*), a weed that is both distributed across China and associated with winter crops, has evolved resistance to acetyl-CoA carboxylase (ACCase) herbicides, but the resistance mechanism remains unclear. The goal of this study was to analyze the transcriptome between resistant and sensitive populations of *P. fugax* at the flowering stage.

**Results:**

Populations resistant and susceptible to clodinafop-propargyl showed distinct transcriptome profiles. A total of 206,041 unigenes were identified; 165,901 unique sequences were annotated using BLASTX alignment databases. Among them, 5904 unigenes were classified into 58 transcription factor families. Nine families were related to the regulation of plant growth and development and to stress responses. Twelve unigenes were differentially expressed between the clodinafop-propargyl-sensitive and clodinafop-propargyl-resistant populations at the early flowering stage; among those unigenes, three belonged to the ABI3VP1, BHLH, and GRAS families, while the remaining nine belonged to the MADS family. Compared with the clodinafop-propargyl-sensitive plants, the resistant plants exhibited different expression pattern of these 12 unigenes.

**Conclusion:**

This study identified differentially expressed unigenes related to ACCase-resistant *P. fugax* and thus provides a genomic resource for understanding the molecular basis of early flowering.

**Electronic supplementary material:**

The online version of this article (10.1186/s12864-017-4324-z) contains supplementary material, which is available to authorized users.

## Background

Common annual Asia minor bluegrass (*Polypogon fugax*) is a weed that is both distributed across China and associated with winter crops. This weed has evolved resistance to clodinafop-propargyl, an acetyl-CoA carboxylase (ACCase) herbicide [1]. The mechanisms of herbicide resistance have been intensively studied in the past twenty years and at least three have been identified: 1) target site change; 2) closure or translocation of herbicides; and 3) alteration in the rate of herbicide metabolism [2–4]. Nevertheless, these three mechanisms alone often fail to explain the development of herbicide resistance from an evolutionary and ecological perspective [5]. Herbicide resistance will increase the fitness of resistant individuals and hence their ability to produce the next generation. Identifying which biological characteristics play a major role on fitness and interactions with environmental factors is essential for predicting herbicide resistance.

It is generally believed that the initial occurrence of major resistance genes in weed populations is the main factor that influences the dynamic evolution of a resistance under herbicide selection [6]. In *Lolium rigidum*, carrying the common Leu-1781 in ACCase affects the fitness of resistant mutants, and the germination rate of the resistant biotype is lower than that of the sensitive biotype [7]. To palliate this lower germination rate, the mutant *Setaria viridis* produces more seeds than the sensitive population [8]. In addition, herbicide-resistant *Setaria* flowers earlier than the susceptible population, with more tillers and panicle, and with lighter seeds [9]. These mechanisms contribute to a more important spread of the resistant populations compared with susceptible ones.

Stress-induced flowering has recently received increased attention. Early flowering and seed setting of resistant plants allow the resistant seeds better access to resources [9]. Photoperiodic flowering and vernalization have been well characterized, and the regulatory mechanisms are well known [10–12]. In addition, flowering physiologists had reported that plants tend to flower when grown under unsuitable conditions, indicating that stress is a flower-inducing factor [13–16]. Stress-induced flowering is now considered as the third category of flowering responses alongside regulated autonomous flowering and environment-induced flowering [17].

Nevertheless, the mechanisms underlying early flowering remain poorly understood, particularly among herbicide-resistant weeds. Stress-induced flowering changes the life cycle and might alter fitness. In addition, stress adaptation extends to the evolution of the flowering characteristics [17]. Transcription factors are proteins regulating gene expression and specific transcription factors selectively regulate the transcriptional expression of specific genes. Therefore, in the present study, we aimed to investigate the transcription factors that regulate plant flowering in order to elucidate the relationship between early flowering and selection pressure (herbicide application) of ACCase-resistant *P. fugax,* and to identify candidate genes responsible for early flowering in resistant plants.

## Methods

### Plants

The seeds of a putative resistant population of *P. fugax* (RP population) were collected from Qingsheng County (29° 54′ 1″ N, 103° 48′ 57″ E), Sichuan Province, China, where clodinafop-propargyl has been used for more than five years and has failed to control *P. fugax* growth. A sensitive population of *P. fugax* (susceptible plant (SP) population) was sampled from a non-cultivated area in Xichang City, Sichuan Province (27° 50′ 56″ N, 102° 15′ 53″ E). The plants were collected without permissions being sought for the nature of scientific research according to the law of the People’s Republic of China.

Because gene expression can differ due to genetic background, genetically homogenized plant material was generated by controlled pairings to narrow the difference. In brief, F1 plants were transplanted to individual 1-L pots in a greenhouse that has an 18/15 °C day/night temperature and a 14-h photoperiod. At the four-tiller stage, the plants were subjected to vegetative propagation: all individual tillers of each plant were separated and transplanted to individual pots. Four clones were therefore obtained. At the three-leaf growth stage, ACCase herbicide was applied. The herbicide sensitivity of each F1 plant was assessed by spraying with clodinafop-propargyl (45 g. active ingredient (a.i.)/ha). Sensitive and resistant F1 plants were then crossed, yielding an F2 population. Visual phenotype rating of the F2 plants was carried out by clodinafop-propargyl selection. The F2 plants with contrasting phenotypes were selected as resistant and sensitive plants. And the F2 generation was used for transcriptome sequencing.

### Sample collection

RPs and SPs (*n* = 18/group) at the seedling stage (3-leaf stage) were selected, and treated with clodinafop-propargyl (R: 45 g.a.i./ha; S: 0.2 g.a.i/ha) (*n* = 9 plants/group-); the remaining plants were treated with an equal volume of water. After 72 h, the aerial parts were collected (*n* = 3 plants/group) and immediately cryopreserved in liquid nitrogen. Afterward, samples were taken at the tillering (four tillers, *n* = 3 plants/group) and flowering (early flowering, *n* = 3 plants/group) stages in the same manner and stored in liquid nitrogen.

### Genomic library construction and sequencing

The total RNA was extracted using TRIzol (Invitrogen Inc., Carlsbad, CA, USA) and DNase I (Takara, Otsu, Japan) in accordance with the manufacturer’s instructions. Magnetic beads with oligo (dT) (Takara, Otsu, Japan) were used to isolate mRNA, and the mRNA was mixed together with fragmentation buffer by a ThermoMixer (Thermo Fisher Scientific, Waltham, MA, USA), breaking the mRNA into short fragments. cDNA was synthesized using the RevertAid First Strand cDNA Synthesis Kit (Fermentas, Thermo Fisher Scientific, Waltham, MA, USA) in accordance with the manufacturer’s instructions. Short fragments were purified and resolved with EB buffer (10 mM Tris·Cl, pH 8.5) to repair their ends by the addition of a single adenine nucleotide. The short fragments were then connected with adaptors (BGI, Beijing, China). Suitable fragments were selected as templates for PCR amplification. The constructed sample library was quantified and characterized using an Agilent 2100 Bioanalyzer (Agilent Technologies, Santa Clara, CA, USA) and an ABI StepOnePlus Real-Time PCR system (Applied Biosystems, Foster City, CA, USA) and then sequenced using a HiSeq 4000 system (Illumina, Inc., San Diego, CA, USA).

### Sequencing and de novo assembly

To obtain high-quality clean reads for de novo assembly, the raw reads were generated from transcriptome sequencing in accordance with the following steps. First, the adaptor sequences were removed. Then, reads with more than 5% of unknown nucleotides were removed and reads with more than 50% of low-quality bases (base quality ≤ 20) were discarded. The clean reads that remained were assembled into unigenes using the Trinity software with an optimized K-mer length of 25 for de novo assembly, as previously published [18]. The expression of unigenes was calculated via the reads per kb per million reads (RPKM, ≥ 0.5), which is a general method of quantifying gene expression from RNA sequencing data by normalizing for total read length and the number of sequencing reads [19].

### Data analysis

Genes expressed at different levels in RPs and SPs (i.e., differentially expressed genes (DEGs)) were subjected to Gene Ontology (GO) functional analysis and Kyoto Encyclopedia of Genes and Genomes (KEGG) pathway analysis. We used getorf software (http://emboss.bioinformatics.nl/cgi-bin/emboss/getorf) to identify the open reading frame (ORF) of each unigene. Then, the ORFs were aligned to the transcription factor (TF) domains using hmmsearch software (http://hmmer.org/) [20].

The false discovery rate (FDR) statistical method was used in multiple hypothesis testing to correct the *P*-value. A smaller FDR and larger ratio indicate a greater difference in the expression level between two samples. In this analysis, we chose samples with an FDR ≤ 0.001 and a ratio greater than 2.

### Differential gene expression analysis

Gene expression levels were estimated using RSEM for each sample, as previously described [21]. Clean reads were mapped back onto the assembled transcriptome, and read count for each gene was obtained from the mapping results and normalized as FPKM (fragments per kilobase of transcript per million mapped reads). Differential expression analysis of the two groups was performed using the DESeq 2. The resulting *P*-values were adjusted using the Benjamini and Hochberg’s approach for controlling the false discovery rate. Genes with an adjusted P-value < 0.05 (fold change ≥ 2) found by DESeq were determined as being differentially expressed. Differentially expressed genes (DEGs) were analyzed by GO and KEGG enrichment analysis.

### GO and KEGG enrichment analyses

All DEGs were mapped to terms in the GO database (http://www.geneontology.org//) and the number of genes corresponding to each GO term was calculated. We established a gene list and gene numbers for each GO term and then used a hypergeometric test to identify DEG GO terms whose enrichment was significantly greater than that in the genome background.

The KEGG pathway database contains networks of molecular interactions in cells and variants specific to particular organisms. We used pathway enrichment analysis to identify DEG metabolic pathways or signal transduction pathways whose enrichment was significantly greater than that in the whole genomic background. After multiple corrections, we selected pathways with an FDR value ≤ 0.001 to represent pathways significantly enriched in DEGs.

### Real-time PCR

The total RNA was extracted as described above and cDNA was synthesized using an M-MLV Rtase Kit (Thermo Fisher Scientific, Waltham, MA, USA) in accordance with the manufacturer’s instructions. The qRT-PCR mix (25 μl) contained 12.5 μl of SYBR Green Mix (Thermo Fisher Scientific, Waltham, MA, USA), 0.5 μl of each primer (10 μM), 2 μl of cDNA, and 9.5 μl of RNase-free water. The reaction was performed on an ABI 7300 real-time PCR system (Applied Biosystems, Foster City, CA, USA). The qRT-PCR program consisted of 95 °C for 10 min, 40 cycles of 95 °C for 15 s and 60 °C for 45 s, and finally 60 °C for 15 s. *EF1* was used as a reference gene for normalization. GraphPad Prism 5 software (GraphPad Software, Inc., La Jolla, CA, USA) was used for data analysis. Expression was calculated in accordance with the 2^-ΔΔCt^ method. Each experiment was repeated at least three times and consisted of three replicates. Primer sequences are listed in Additional file 1: Table S1.

### Data access

The raw reads have been deposited in the NCBI Sequence Read Archive (SRA) database (BioProject PRJNA385696, SRP106591). The data are available at https://trace.ncbi.nlm.nih.gov/Traces/sra_sub/sub.cgi?acc=SRP106591&focus=SRP106591&from=list&action=show:STUDY.

## Results

### *Polypogon fugax* Transcriptome sequencing and data assembly

Compared with *P. fugax* SPs, RPs flowered 10-15 days earlier, and their inflorescence was morphologically altered, RPs produced 1.9 times more seed than did SPs Additional file 2: Figure S1). Therefore, the transcriptome of SPs and RPs were compared to explore the potential genes involved in this process.

Strand-specific RNA-Seq was applied to assess RNAs from three pairs of RPs and SPs at the seedling, tillering, and flowering stages with or without clodinafop-propargyl treatment (seedling stage) to comprehensively identify the unigenes associated with herbicide resistance.

The sequencing reads containing low-quality, adaptor-contaminated, or high contents of unknown base (N) reads were removed before downstream analyses. Afterward, 150-base single-end sequence raw reads were subjected to quality control using the Phred scaled quality score. Overall, 1.39 billion raw reads and 1.07 billion clean reads (average clean read ratio of 77.05%) were obtained; 92.8% of the clean reads had a quality score ≥ 30, and 97.7% of the clean reads were quality filtered and matched the Illumina’s quality requirements. Read quality metrics after filtering are shown in Additional file 3: Table S2. De novo assembly of the 150-base reads yielded 206,041 unique sequences ranging from 300 to 3000 nt in length (Table 1) (including 14,166 unigenes with sequences of up to 3000 nt in length) Additional file 3: Table S3). The length distribution of the assembled contigs is shown in Additional file 4: Figure S2. Among the detected 206,041 unigenes, 165,901 unique sequences were annotated based on BLASTX alignment (E-value <0.00001) searches of seven databases: the NCBI non-redundant (NR), NCBI non-redundant nucleotide (NT), Swiss-Prot protein, KEGG, Cluster of Orthologous Groups of proteins (COG), InterPro protein, and GO databases (Additional file 3: Table S4). The 153,591 unique sequences were annotated by reference to the NR database, and then compared to those encoded in the genomes of all grass (*Poaceae*) species whose genome is fully sequenced, i.e., *Brachypodium distachyon*, *Hordeum vulgare*, *Aegilops tauschii,* and *Triticum urartu*. (Additional file 5: Figure S3).

**Table 1:** Summary of *Polypogon fugax* transcriptome sequencing, assembly, and annotation

	Reference transcriptome
Total clean reads	275,659,060
Assembled unigenes	206,041
Average read length	1337
N50 contig size	1851
N90 contig size	705
Annotation in NR	153,591
Annotation in NT	145,977
Annotation in KO	121,526
Annotation in SwissProt	111,290
Annotation in GO	80,312
Annotation in COG	74,434
Annotation in Interpro	93,000

NR, NCBI non-redundant protein sequences database

NT, NCBI nucleotide sequences database

KO, KEGG (Kyoto Encyclopedia of Genes and Genomes) Orthology database

GO, Gene Ontology

COG, Clusters of Orthologous Groups of Proteins

### Annotation of assembled unigenes

To further examine the integrity and effectiveness of the annotation process, the number of unigenes (that have NR matches) with a COG classification was calculated. A total of 74,434 unigenes were identified with a COG classification (Additional file 3: Table S5). Among the 25 COG categories, the cluster of “General function prediction only” had the highest number (21,107, 28.36%), followed by “Transcription” (15,595, 20.95%), and “Function unknown” (14,816, 19.90%). Categories of “Extracellular structures” (86, 0.001%) and “Nuclear structure” (11, 1.48 e^−4^) had the fewest matching genes (Additional file 6: Figure S4).

**Fig. 1:**
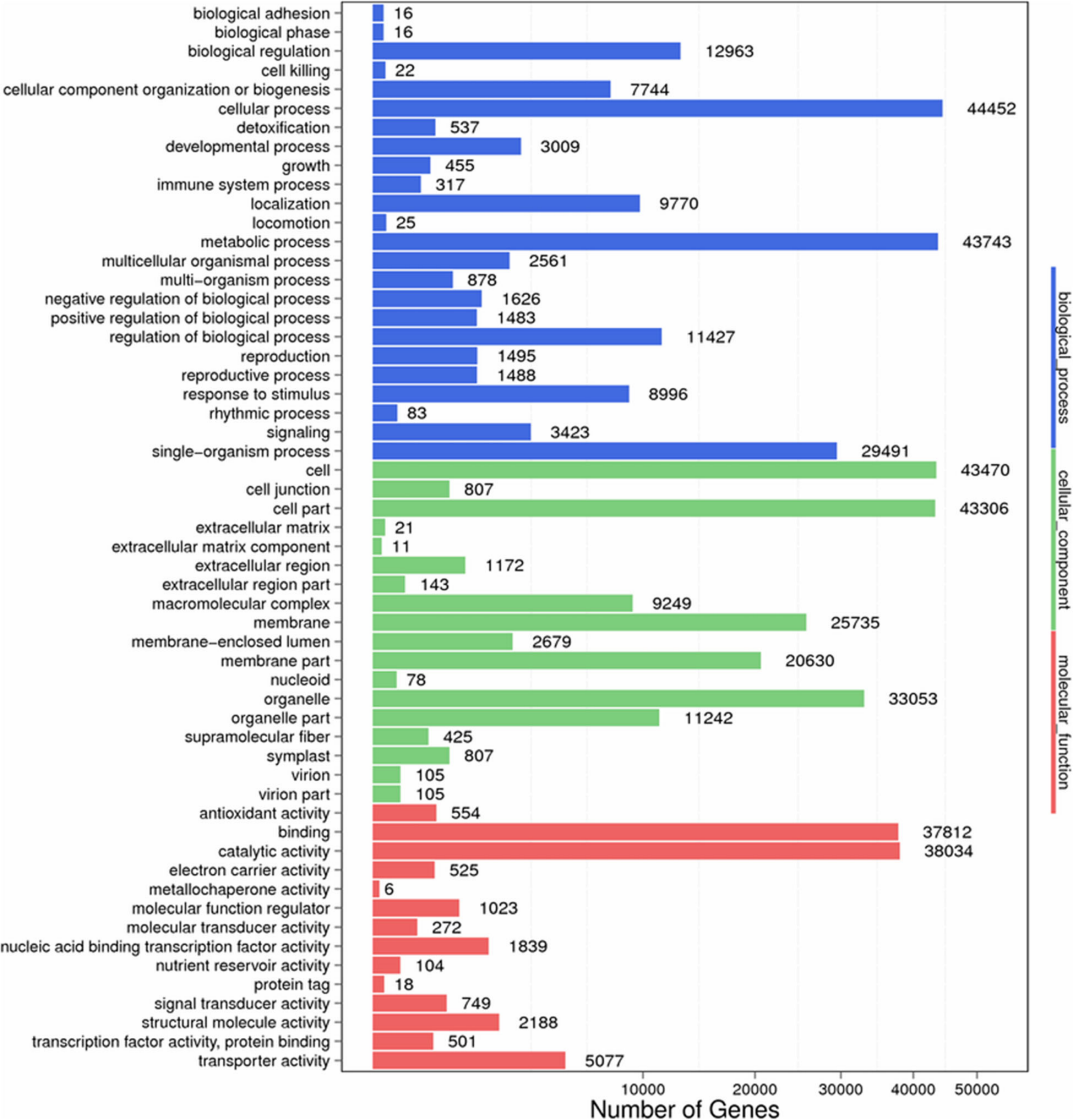
Histogram of GO classification. Based on the homologous genes, 80,312 sequences from all unigenes of 36 *P. fugax* libraries were categorized into 56 GO terms comprising three domains: biological process, cellular component, and molecular function. The majority of the terms were categorized as “cellular process”, “metabolic process”, “cell”, and “cell part”. A high percentage of genes were also assigned to “binding”, “catalytic activity”, “organelle”, and “membrane”, and as well as “biological regulation”, “development process”, “transporter activity”, and “reproductive process”

GO and KEGG enrichment analyses were used to classify the functions of the predicted *P. fugax* unigenes. Based on homologous genes, 80,312 sequences (Additional file 3: Table S6) from all unigenes of 36 *P. fugax* libraries were categorized into 56 GO terms comprising three domains: biological process, cellular component, and molecular function (Fig. 1). Most were categorized in “cellular process”, “metabolic process”, “cell”, and “cell part”. A high percentage of genes were also assigned to “binding”, “catalytic activity”, “organelle”, and “membrane” as well as “biological regulation”, “development process”, “transporter activity”, and “reproductive process” (Fig. 1).

**Fig. 2:**
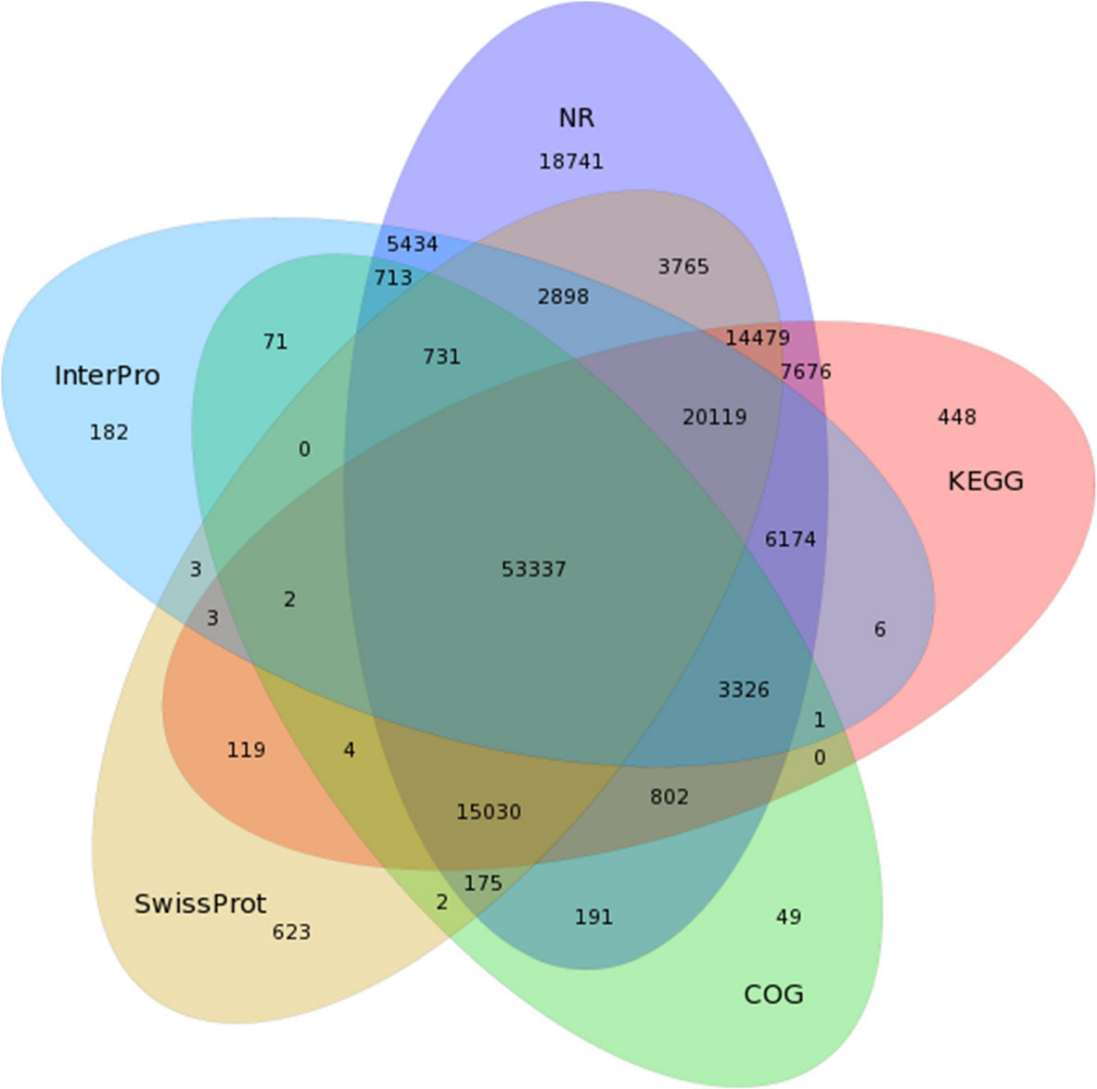
Venn diagram of the NR, COG, KEGG, SwissProt, and InterPro databases. A total of 53,337 annotated unigenes between the NR, COG, KEGG, SwissProt, and InterPro databases were identified by Venn diagram

There were 121,526 unigenes that mapped onto KEGG pathways (Additional file 3: Table S7). A total of 53,337 annotated unigenes between NR, COG, KEGG, SwissProt, and InterPro databases were identified with Venn diagrams (Fig. 2).

### TF prediction of assembled unigenes

**Fig. 3:**
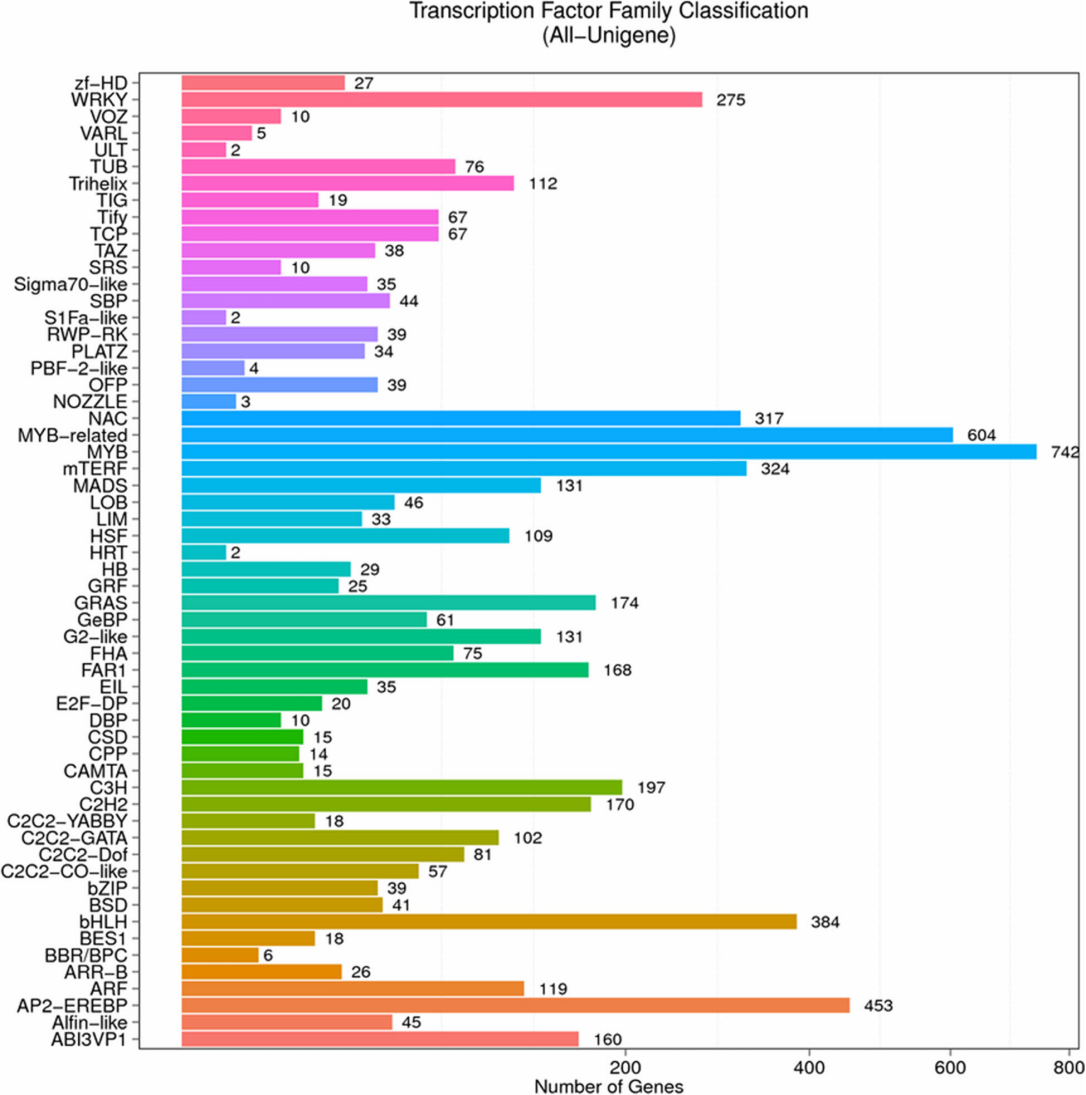
TF family classification of unigenes. In total, 5904 unigenes were classified into 58 TF families. Among these families, the MYB family represented the highest number (742, 12.57%), followed by the MYB-related family (604, 10.23%) and the AP2-EREBP family (453, 7.67%). Genes of the MADS-box family, which are associated with plant development and adversity responses, were also identified (131, 0.02%). The X axis represents the number of unigenes. The Y axis represents the TF family. “All-Unigene” indicates that the unigenes were those assembled from the 36 samples of *Polypogon fugax*

Next, we studied unigenes that encoded TFs. The list of unigenes that encode TFs is shown in Additional file 3: Table S8. We also performed TF family classification, and found that 5904 unigenes were classified into 58 TF families (Fig. 3). Among those families, the MYB family had the highest number (742, 12.57%), followed by the MYB-related family (604, 10.23%) and the AP2-EREBP family (453, 7.67%). Genes of the MADS-box family were also found (131, 0.02% %); these genes are associated with plant development and adversity responses.

### qRT-PCR validation of *P. fugax* expression data

To verify the gene expression patterns, qRT-PCR analyses were performed on Unigene12462, CL1441.Contig20, CL21112.Contig3, CL4600.Contig9 and CL4600. Contig2, CL12188.Contig2, and *EF1* served as candidate reference genes for RT-PCR normalization.

**Fig. 4:**
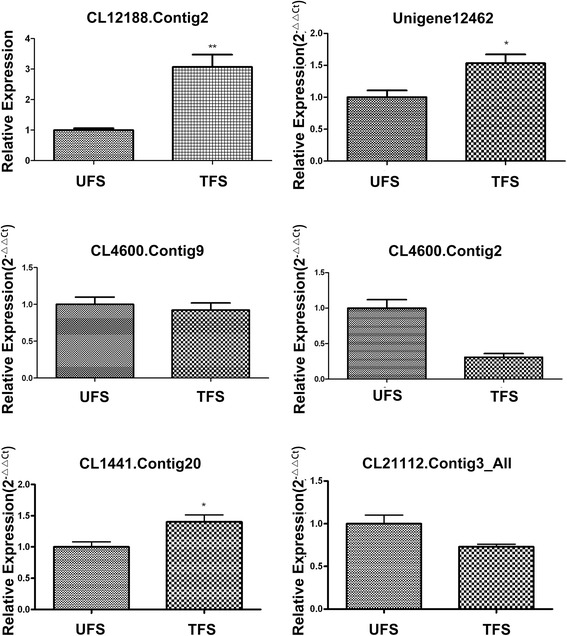
qRT-PCR validation of RNA-Seq results. qRT-PCR analysis of six randomly selected genes was conducted to confirm the expression patterns indicated by sequencing. The expression of CL12188.Contig2, Unigene12462, and CL1441.Contig20 was higher in the TFS than in the UFS. CL4600.Contig2 and CL21112.Contig3 was higher in the UFS than in the TFS. These results confirmed the reliability of the data. The error bars represent the standard error of the mean. U: untreated; T: treated; F: flowering stage; S: sensitive population; R: resistant population. *significant difference p>0.05, **significant difference 0.01<P<0.05.

The expression of CL12188.Contig2, Unigene12462, and CL1441.Contig20 was higher in the treated sensitive population when flowering (TFS) than in the untreated sensitive population when flowering (UFS). CL4600.Contig2 and CL21112.Contig3 were higher in the UFS than in the TFS. These results confirmed the reliability of the data (Fig. 4).

### Functional analysis of DEGs

**Fig. 5:**
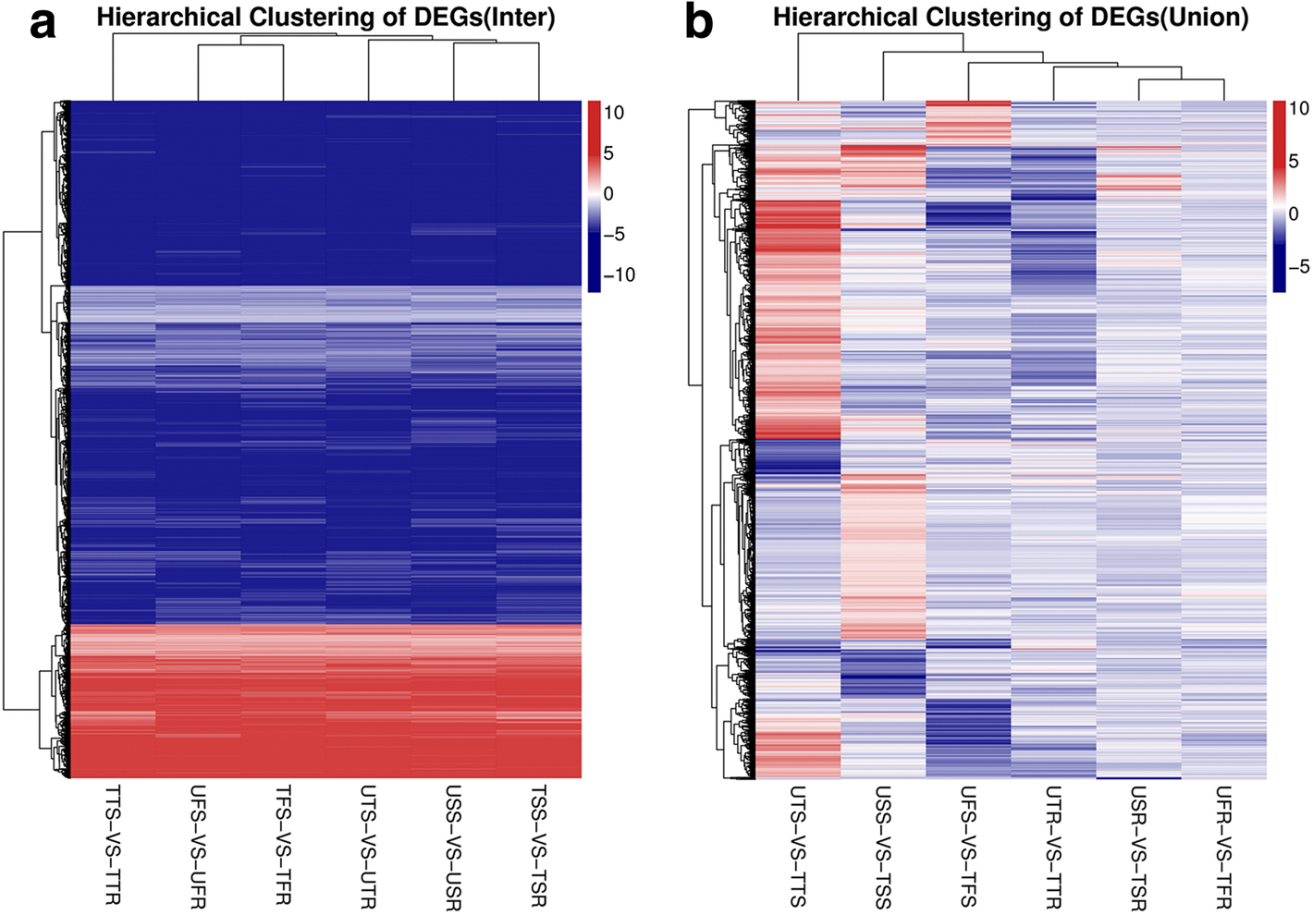
Cluster analysis of DEGs. Cluster analyses of inter (**a**) and inner (**b**) group comparisons were analyzed by heat maps. Fifty-eight TF families were ultimately predicted, nine families were related to the regulation of plant growth and development and stress responses. We analyzed the different expression values of the TF families (log2-fold change) of the different populations (resistant vs. sensitive) under the same treatment at the same time, including the MYB family, MYB-related family, MADS family, NAC family, mTERF family, ABI3VP1 family, AP2-EREBP family, bHLH family, and GRAS family

To screen the flowering regulatory genes related to resistance, we analyzed DEGs among the seedling, tillering, and flowering stages under different treatments. The genes at the seedling and tillering stages served as a background to identify the specific DEGs at the flowering stage. Cluster analysis was used to compare DEGs, and the parameter was the log2 ratio of gene expression in the difference comparison scheme. The Euclidean distance (calculation of gene distance) referred to genes differentially expressed among all groups. Inter- and inner-group comparisons were performed by the same methods (Fig. 5a-b). Fifty-eight TF families were ultimately predicted, and nine families were related to the regulation of plant growth and development and stress response. We analyzed the different expression values of the TF families (log2-fold change) of the different populations (resistant vs. sensitive) under the same treatment at the same time, including the MYB (Additional file 3: Table S9), MYB-related (Additional file 3: Table S10), MADS (Additional file 3: Table S11), NAC (Additional file 3: Table S12), mTERF (Additional file 3: Table S13), ABI3VP1 (Additional file 3: Table S14), AP2-EREBP (Additional file 3: Table S15), bHLH (Additional file 3: Table S16), and GRAS (Additional file 3: Table S17) families.

**Fig. 6:**
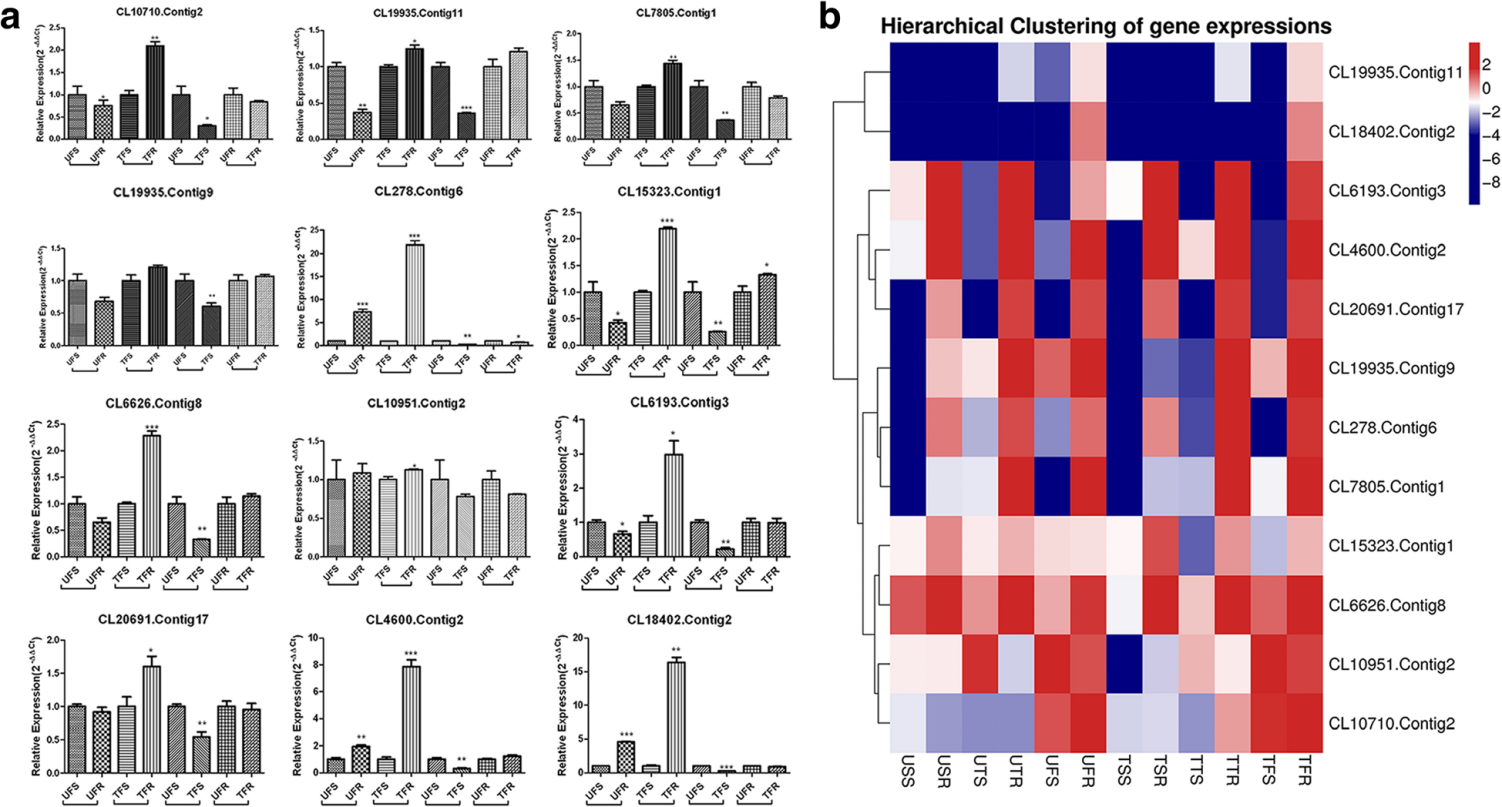
**a** Comparison of the expression levels of 12 unigenes in differently treated samples and groups at the flowering stage. Three replicates were performed for each of the three biological replicates. The error bars represent the standard error of the mean. U: untreated; T: treated; F: flowering stage; S: sensitive population; R: resistant population. *significant difference p>0.05, **significant difference 0.01<P<0.05, ***significant difference P<0.01. **b** Heat map analysis of the 12 unigenes

For the DEGs in the nine abovementioned TF families, the screening criteria were as follows: 1) the expression levels of the RPs at the three stages were all higher than those of the SPs regardless of herbicide application; 2) the gene expression of the RPs was higher than that of the SPs after spraying; 3) the DEGs were expressed only at the flowering stage and not at the seedling or tillering stage; and 4) the expression in the sprayed resistant population was higher than that in the sensitive population only at the flowering stage (Additional file 3: Tables S9-S17, sheet 1). Afterward, a total of 30 candidate genes were selected for screening resistance related flowering-regulated genes. qRT-PCR was then carried out to verify the expression of these 30 genes in four samples: the UFS, untreated resistant population at the flowering stage(UFR), TFS, and treated resistant population at the flowering stage(TFR). Twelve DEGs were related to the regulation of plant development, flowering, and stress response (Fig. 6a-b, Table 2); the remaining 18 genes were false positives (data not shown). The ABI3VP1, BHLH, and GRAS families each had one gene (CL18402.Contig2, CL6193.Contig3, and CL20691.Contig17), and the other nine genes belonged to the MADSbox family. The expression of four genes (CL4600.contig2, CL278.contig6, CL10951.contig2, and CL18402.contig2) was higher in the RPs than in the SPs under herbicide and water treatments. The expression of eight genes (CL15323.contig1, CL6626.contig8, CL10710.contig2, CL19935.contig11, CL7805.contig1, CL19935.contig11, CL6193.contig3, and CL20691.contig17) was slightly lower in the RPs than in the SPs under water treatment, but their expression levels increased rapidly after herbicide application and, consequently, were significantly higher than those of the SPs. Interspecific comparisons showed that the expression of 12 unigenes in the RPs was higher than that in the SPs under herbicide selective pressure, suggesting that these genes in the RPs likely promote reproductive growth (flowering and fruiting) under stress conditions: this phenomenon constitutes an unknown resistance mechanism (Fig. 6a).

**Table 2:** Differentially expressed genes (DEGs) as candidate transcription factors (TFs) between the susceptible plants (SPs) and resistant plants (RPs) of *Polypogon fugax* at the early flowering stage^a^

Unigene ID	Contig length	Gene family	Gene annotations	ID in database	Log_2_Ratio (UFR/UFS)	P_adj_	Log_2_ ratio (TFR/TFS)	P_adj_
CL10710.contig2	1396	MADS	MIKC-type MADS-box transcription factor WM29B [*Triticum aestivum*]	CAM59077.1	2.44949	7.16E-07	2.08657	0.00886
CL19935.contig11	1314	MADS	VRN1 [*Festuca arundinacea*]	ACN81330.1	1.41482	0.65251	3.95796	0.05880
CL7805.contig1	1423	MADS	MADS3 [*Lolium perenne*]	AAO45875.1	6.75310	5.83E-09	4.31204	5.94E-05
CL19935.contig9	1496	MADS	MADS-box protein 1 [*Lolium temulentum*]	AAD10625.1	2.71062	1.03E-07	3.93220	1.90E-06
CL278.contig6	814	MADS	predicted protein [*Hordeum vulgare subsp. vulgare*]	BAK01668.1	2.79704	0.034962	4.56666	0.00195
CL15323.contig1	985	MADS	MADS-box transcription factor 42 [*Brachypodium distachyon*]	AIG21850.1	1.60674	0.38702	1.88285	0.20711
CL6626.contig8	1089	MADS	unnamed protein product [Triticum aestivum]	CDM85805.1	1.49045	0.17262	1.01250	0.38150
CL10951.contig2	1176	MADS	MADS-box transcription factor WM21B[Triticum aestivum]	AM502888.1	−1.65321	0.02961	−1.30507	0.22610
CL4600.contig2	1233	MADS	VRT2 [Festuca arundinacea]	ADK55060.1	4.11115	0.00056	5.05851	0.00019
CL19114.Contig2	1056	MADS	MADS-box transcription factor 31 [*Aegilops tauschii*]	EMT17017.1	5.10271	7.61E-05	4.99548	0.00040
CL3687.Contig1	1187	MYB	hypothetical protein OsJ_36546 [*Oryza sativa* Japonica Group]	EAZ20907.1	6.04532	0.00030	5.34815	0.00197
Unigene19990	1015	MYB	Myb-related protein MYBAS2 [Aegilops tauschii]	EMT18692.1	4.06133	1.33E-07	4.23625	9.57E-05
Unigene7488	1353	MYB	hypothetical protein SETIT_ 017851 mg [*Setaria italica*]	KQL30198.1	5.44547	0.00105	4.25490	0.01766
Unigene58296	2044	BHLH	predicted protein [Hordeum vulgare subsp. vulgare]	BAJ85853.1	3.22304	0.03804	5.82932	0.00086
CL9764.Contig4	1172	BHLH	hypothetical protein F775_42459 [Aegilops tauschii]	EMT14257.1	3.79240	0.01288	4.66703	0.01675
CL20691.Contig14	2701	GRAS	predicted protein [Hordeum vulgare subsp. vulgare]	BAJ89210.1	3.77490	5.83E-05	5.10209	0.00015
CL3809.Contig22	2464	GRAS	predicted protein [Hordeum vulgare subsp. vulgare]	BAJ97549.1	3.44933	3.96E-05	3.64527	0.00743
CL18402.contig2	1556	ABI3VP1	B3 domain-containing protein Os01g0234100-like [Brachypodium distachyon]	XP_010240699.1	5.06971	0.00733	4.55668	0.02181
CL12188.Contig2	4196	ABI3VP1	hypothetical protein OsI_35443 [Oryza sativa Indica Group]	EEC67837.1	5.77226	7.86E-07	4.04356	5.17E-05
Unigene13022	1367	ABI3VP1	unnamed protein product [Triticum aestivum]	CDM85878.1	5.75417	0.00076	5.08982	0.00602
Unigene51942	1567	AP2-EREBP	hypothetical protein BRADI_ 1 g46690 [Brachypodium distachyon]	KQK19167.1	6.70426	1.01E-23	7.43238	3.86E-14
Unigene12462	2057	AP2-EREBP	DRF-like transcription factor DRFL2a [Triticum aestivum]	ABC74510.1	9.51535	9.86E-13	9.17289	3.86E-11
CL8782.Contig4	1181	AP2-EREBP	ethylene-responsive transcription factor-like protein At4g13040 [Brachypodium distachyon]	XP_003574993.2	−1.89557	0.13175	4.77569	0.01210
CL1441.Contig20	1766	NAC	predicted protein [Hordeum vulgare subsp. vulgare]	BAK08125.1	8.03481	2.09E-08	6.60687	4.59E-08
Unigene25784	1076	NAC	hypothetical protein MNEG_0811 [Monoraphidium neglectum]	KIZ07145.1	6.80800	6.75E-06	6.49068	1.84E-05
CL21112.Contig3	1951	NAC	NAC transcription factor [Hordeum vulgare subsp. vulgare]	CBZ41156.1	4.96265	8.40E-08	7.80505	1.52E-07
Unigene31436	2102	NAC	predicted protein [Hordeum vulgare subsp. vulgare]	BAJ93083.1	6.13811	2.79E-07	5.17007	8.91E-08

^a^Limitations of all significantly different expressed genes between the susceptible plants (SP) and resistant plants (RP) of *Polypogon fugax* are based on P_adj_ < 1 and the absolute value of log_2_ ratio ≥ 1. The log_2_ ratio indicated the change of gene expression; a positive number means up-regulation and a negative one means down-regulatio

Under all treatment conditions, expression levels of the 12 unigenes were significantly higher in the UFS than in the TFS, which means that the expression of these genes in SPs was inhibited by herbicide selection. In the RPs, the expression of four unigenes (CL6193.contig3, CL20691.contig17, CL18402.contig2, and CL19935. contig9) in the UFR did not differ from that in the TFR (Fig. 6a). The expression of four unigenes (CL4600.contig2, CL19935.contig11, CL15323.contig1, and CL6626.contig8) was slightly lower in the UFR than in the TFR (Fig. 6a), and the expression of four unigenes (CL10710.contig2, CL7805.contig1, CL278.contig6, and CL10951.contig2) was slightly higher in the UFR than in the TFR (Fig. 6a). The results of the intraspecific comparison showed that herbicide selection pressure did not significantly influence the expression of these genes in the RPs (Fig. 6a). The results indicate that these genes have genetically adapted in RPs, while herbicides did not affect the growth and development of those plants.

## Discussion

In this study, a *P. fugax* population resistant to clodinafop-propargyl and a susceptible population were selected. The RPs flowered 10-15 days earlier than did the SPs, but the mechanisms remain unclear. The goal of this study was to establish a resource to study transcriptomic patterns in *P. fugax* resistant or sensitive to clodinafop-propargyl in the absence or presence of herbicide and at different stages (seeding, tillering, and flowering), using experimental conditions as similar as possible to field conditions. The results revealed DEGs related to clodinafop-propargyl resistance in *P. fugax*. The assembled, annotated transcriptomes provide a genomic resource for understanding the molecular basis of *P. fugax* herbicide resistance.

Because there is no genomic resource for *P. fugax*, the Illumina technology was selected for sequencing, as it is the technology of choice for de novo transcriptome deep sequencing and assembly when a reference genome is absent [22]. Three replicates of 12 samples were sequenced by an Illumina HiSeq 4000 in this study, generating approximately 160.52 Gb bases in total. After discarding improper sequences, 206,041 unigenes were obtained; the total length, average length, N50, and GC content of these unigenes were 275,659,060 bp, 1337 bp, 1851 bp, and 51.51%, respectively. The N50 size of the contigs in this study was 1851 bp, which is higher than that recently obtained for plant de novo transcriptome assemblies based on Illumina sequence reads [23, 24]. The average contig size was 1337 bp, which matched the average length of gene coding sequences in grasses (1000 to 1300 bp) [25]. The use of functional annotation results revealed 152,966 coding DNA sequences (CDS), ESTScan (v3.0.2) was then used to predict the remaining unigenes, after which 11,716 additional CDS were obtained. Given that only 48 nucleotide sequences from *P. fugax* had been deposited in GenBank on May 22nd, 2017, our work tremendously increased the sequence data available for *P. fugax*.

The predicted peptide content of the *P. fugax* transcriptome was compared to that of five fully sequenced grass genomes. The five grass species belong to three major subfamilies of the *Poaceae*: *Pooideae* (*Brachypodium distachyon* and *H. vulgare*), *Panicoideae* (*Zea mays* and *Sorghum bicolor*), and *Ehrhartoideae* (*Oryza sativa*) [26]. The five grass genomes shared 58.64% of the identified protein families. These proportions were in agreement with a previous genome-wide study showing that genome peptide contents are largely shared among grass species, including peptide family representations [27]. In addition, good representation of the transcriptome of the aerial parts of *P. fugax* at different stages and confirmation of the relevance of the RNA-Seq-based expression data using qRT-PCR make the sequences a reliable resource for investigating the transcriptomic response to herbicide stress promoting early flowering in *P. fugax*.

Stress-induced flowering is a response to stress and is the ultimate stress adaptation, as plants can survive as a species if they flower and produce seeds under severe stress even when they cannot survive as individuals [17]. To validate the function of the candidate genes with respect to flowering in the resistant population, gene expression at the seedling and tillering stages served as a background. The present study used herbicide selection pressure when the RPs and SPs were about to flower. qRT-PCR was subsequently applied to analyze the candidate gene expression at the flowering stage to understand the relationship and mechanism of flowering and response to herbicide application in *P. fugax*.

The replication and diversity of MADS-box genes may be the factors affecting the morphological diversity of land plants and some angiosperms [28]. This gene family encodes conserved TFs and plays an important role in both vegetative and reproductive development. Among these genes, *APETALA1* (*AP1*) is one of the earliest and most intensively studied genes. In *Arabidopsis*, *AP1* deletion mutations delay flowering time and show a high frequency of floral meristem to inflorescence meristem transformation after flowering. On the other hand, constitutive expression of the *AP1* gene changes the floral bud meristem to a floral meristem, and ectopic expression of the *AP1* gene can significantly promote early flowering [29, 30]. The *AP1* gene in herbaceous [31] and woody [32] plants also plays an important role in the initiation and development of flowers, and constitutive expression of *AP1*-like genes also contributes to early flowering. In contrast to the *AP1* gene, overexpression of some MYB TFs (such as *EPR1* and *AtMYB44*) in *Arabidopsis* leads to delayed flowering time [33, 34].

Table 2 summarizes the differentially expressed TFs identified in the present study. CL4600.Contig2 was annotated to the *JOINTLESS*-like protein (ADK55060.1). *JOINTLESS* (*J*) is a MADS-box gene that belongs in the same clade as the *Arabidopsis* flowering time genes *SHORT VEGETATIVE PHASE* (*SVP*) and *AGAMOUS LIKE 24* (*AGL24*) [35]. Loss of *J* function causes premature termination of flower formation during inflorescence and reversion to a vegetative sympodial growth [36]. In addition, the formation of an inflorescence in tomato requires the interaction of *J* and a target of *SINGLE FLOWER TRUSS* (*SFT*) in the meristem [37].

CL10951.Contig2 was annotated to *SUPPRESSOR OF OVEREXPRESSION OF CONSTANS1* (*SOC1*), which plays an important role in the regulation of flowering by integrating multiple flowering signals in *Arabidopsis thaliana* [38]. In the photoperiod pathway, *SOC1* is regulated by *CONSTANS* (*CO*) through *FLOWERING LOCUS T* (*FT*), causing early flowering [39]. In addition, similar to *SOC1*, *SOC1*-like genes promote flowering when overexpressed, but some are also involved in floral development. Ectopic expression of *UNSHAVEN* (*UNS*), a *SOC1*-like gene of *Petunia* hybrids, leads to ectopic trichome formation on floral organs and the formation of petals into organs that exhibit leaf-like features and an early flowering phenotype [40, 41]. CL10951.Contig2 may be related to the promotion of flowering in *P. fugax* under herbicide stress [42].

*AGAMOUS-like* proteins (CL10710.Contig2, CL19935.Contig11, CL19935. Contig9, CL278.Contig6, and CL7805.Contig1) belong to the *AG* subfamily whose members are involved in the specification of floral reproductive organs and are also required for the normal development of carpels and fruits in *Arabidopsis* and *Gossypium hirsutum* [43] as well as for both drought stress responses [44] and regulation of post-germination growth [45]. *AGAMOUS*-like proteins under herbicide selection pressure were identified in this study, suggesting that these genes may promote early flowering and increased seed yield in resistant *P. fugax* plants.

The remaining three unigenes (CL18402.Contig2, CL6193.Contig3, and CL20691.Contig17) belong to the ABI3VP1, BHLH, and GRAS families, respectively. The genes of these three families play important roles in plant growth and development and stress responses [46–48], but their specific roles in *P. fugax* under herbicide stress need to be studied further.

The present study is not without limitations. Only two cultivars of *P. fugax* were studied, it is possible that cultivars from different regions could yield different results. In addition, transcriptome analysis is limited by available comparative data. It must be stressed that the present study does not provide any mechanistic data, but rather makes available transcriptomic data that could be used for the determination of those mechanisms. Additional studies are necessary to improve our knowledge and understanding of transcriptomics in plants.

## Conclusions

In conclusion, the present study compared ACCase-resistant to ACCase-sensitive *P. fugax*, as RPs flower earlier and yield more seeds. The results revealed nine related resistance genes in the MADS-box family (which regulate flowering), and three genes involved in the regulation of growth and development. These data lay the foundation for the further exploration of the specific functions of these genes and for the study of transcriptomics in grasses.

## Additional files

Additional file 1: Table S1. Primers used for qRT-PCR. (DOC 63 kb)

Additional file 2: Figure S1. Morphological characteristics in resistant (R) and susceptible (S) Asian minor bluegrass plants at different growth stages. (TIFF 1872 kb)

Additional file 3: Table S2. Summary of sequencing reads after filtering. **Table S3.** Quality metrics of unigenes. **Table S4.** Summary of functional annotation result. **Table S5.** All-unigenes with a COG classification. **Table S6.** All-unigenes categorized by GO terns. **Table S7.** Unigenes mapped onto KEGG pathways. **Table S8.** TF prediction results. **Table S9.** MYB family. **Table S10.** MYB-related family. **Table S11.** MADS-box family. **Table S12.** NAC family. **Table S13.** mTERF family. **Table S14.** ABI3VP1 family. **Table S15.** AP2-EREBP family. **Table S16.** bHLH family. **Table S17.** GRAS family. (ZIP 3364 kb)

Additional file 4: Figure S2. Length distribution of unigenes. (TIFF 5378 kb)

Additional file 5: Figure S3. Distribution of annotated species. (TIFF 3199 kb)

Additional file 6: Figure S4. Functional distribution of COG annotation. (TIFF 6509 kb)

## Abbreviations

ACCase: acetyl-CoA carboxylase
COG: Cluster of Orthologous Groups of proteins
DEGs: differently expressed genes
FDR: false discovery rate
GO: Gene Ontology
KEGG: Kyoto Encyclopedia of Genes and Genomes
NR: non-redundant
NT: nucleotide
ORF: open reading frame
RPKM: reads per kilobase per million reads
SRA: Short Read Archive
TFs: transcription factors
