# Supplementary material for: Transcriptomics analysis of the flowering regulatory genes involved in the herbicide resistance of Asia minor bluegrass (Polypogon fugax)
Source: BMC Genomics. 2017 Dec 6;18:953. doi: 10.1186/s12864-017-4324-z (PMC5719899; doi:10.1186/s12864-017-4324-z)
Supplement: Supplementary file 1 — Primers used for qRT-PCR. (DOC 63 kb) [file 12864_2017_4324_MOESM1_ESM.doc]

**Table S1 Primers for qRT-PCR**

| ID | Gene Name | Sequence (5'-3') | Size (bp) |
| --- | --- | --- | --- |
| 1 | Reference gene (*EF1a*) | GAACCTCCCAGGCTGATTGT | 117 |
|  |  | CAAGAGTGAAAGCAAGAAGAGCA |  |
| 2 | CL10710.Contig2 | GGAGTTCTGGGTTCGGGTTA | 104 |
|  |  | CACAGAGGTCGACTAGCACA |  |
| 3 | CL19935.Contig11 | AAGAAGGAGAGGGCATTGCT | 112 |
|  |  | TGGTTTGAGGCTGAGTTTGC |  |
| 4 | CL7805.Contig1 | TCCTCTCCTCCTGCTTCTCT | 100 |
|  |  | CAACAACAAGCTCACCGTCA |  |
| 5 | CL19935.Contig9 | AAGAAGGAGAGGGCATTGCT | 112 |
|  |  | TGGTTTGAGGCTGAGTTTGC |  |
| 6 | CL278.Contig6 | CTCCCTAATCCCCAAGACCC | 106 |
|  |  | GCTCATTTGCCCTGTTTCCA |  |
| 7 | CL15323.Contig1 | GTGAACATGCTGGAAAGGGG | 108 |
|  |  | CCAGCCAAGTAAACCGGATG |  |
| 8 | CL6626.Contig8 | TGGAACACTACCGAGCAACT | 108 |
|  |  | TCAGTCTTCACCCCTGTCAC |  |
| 9 | CL10951.Contig2 | GGACGATCGATACAGGGGTT | 102 |
|  |  | GATACGGAGATGCAAGCGAC |  |
| 10 | CL6193.Contig3 | GTTCCCCTTCCTGCCTTTTG | 132 |
|  |  | CCACTGGATGAACATGCTCG |  |
| 11 | CL20691.Contig17 | ATGAACTCCGCCTTCCTCAA | 113 |
|  |  | TCTCGGCCCCTGAAGAAATT |  |
| 12 | CL18402.Contig2 | TCACCATGTTAAGCGAATCGT | 111 |
|  |  | AGTGTCTCAGTCGCTTCAGT |  |
| 13 | CL4600.Contig2 | CAAACACCGGAGAGAGGCTA | 149 |
|  |  | TTCCCCTCCTCCTTTCGTTC |  |
| 14 | CL12188.contig2 | CCTTTCCTTTCGTGCTGGAC | 100 |
|  |  | GATGCCTCATGTTCCCTCCT |  |
| 15 | Unigene 12462 | GAGGGCTTGGCTAGAGGTAG | 133 |
|  |  | CCAACTCCCCTGGTTTCTCT |  |
| 16 | CL1441.contig20 | CCCAGTCATCCAACCTGAGT | 145 |
|  |  | AAGAAGGCGCTCGTGTTCTA |  |
| 17 | CL2112.contig3 | GACCACCTCTGAAAGCAACC | 100 |
|  |  | CTTGGTTCAGTTTCGTGGCA |  |
