# Supplementary figures and images for: Transcriptomics analysis of the flowering regulatory genes involved in the herbicide resistance of Asia minor bluegrass (Polypogon fugax)
Source: BMC Genomics. 2017 Dec 6;18:953. doi: 10.1186/s12864-017-4324-z (PMC5719899; doi:10.1186/s12864-017-4324-z)

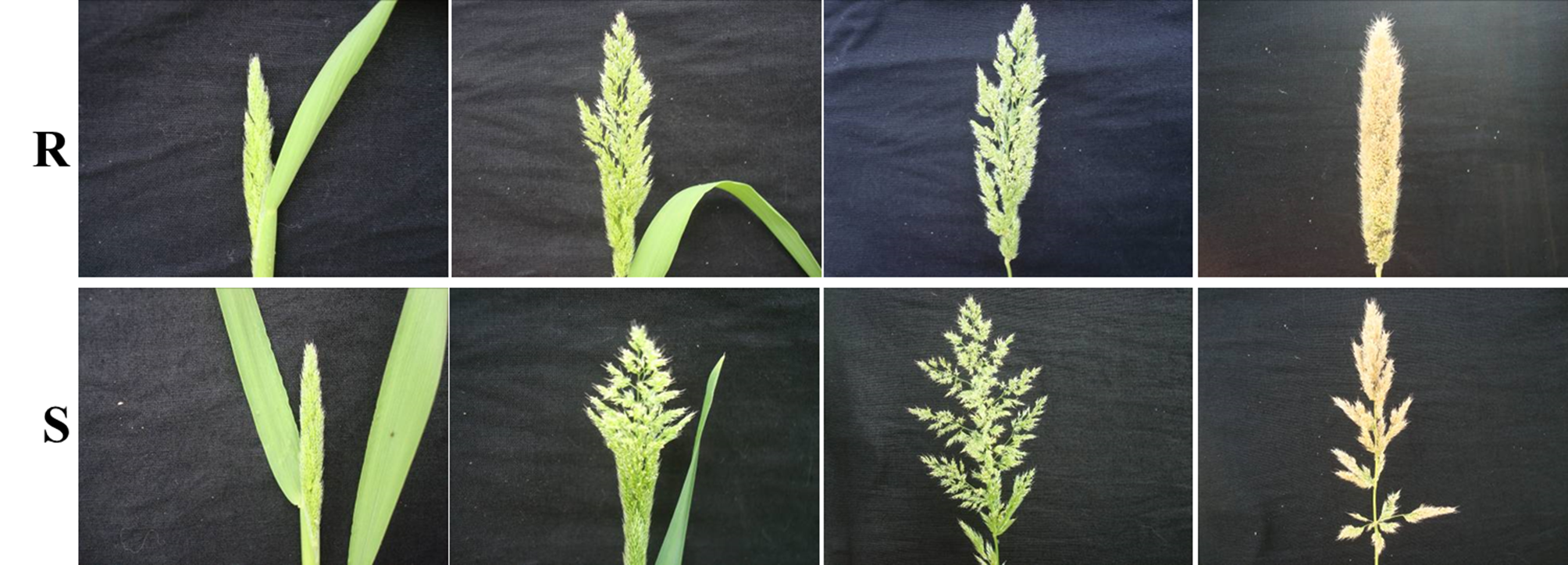

Supplement: Supplementary file 2 — Morphological characteristics in resistant (R) and susceptible (S) Asian minor bluegrass plants at different growth stages. (TIFF 1872 kb) [file 12864_2017_4324_MOESM2_ESM.tif]

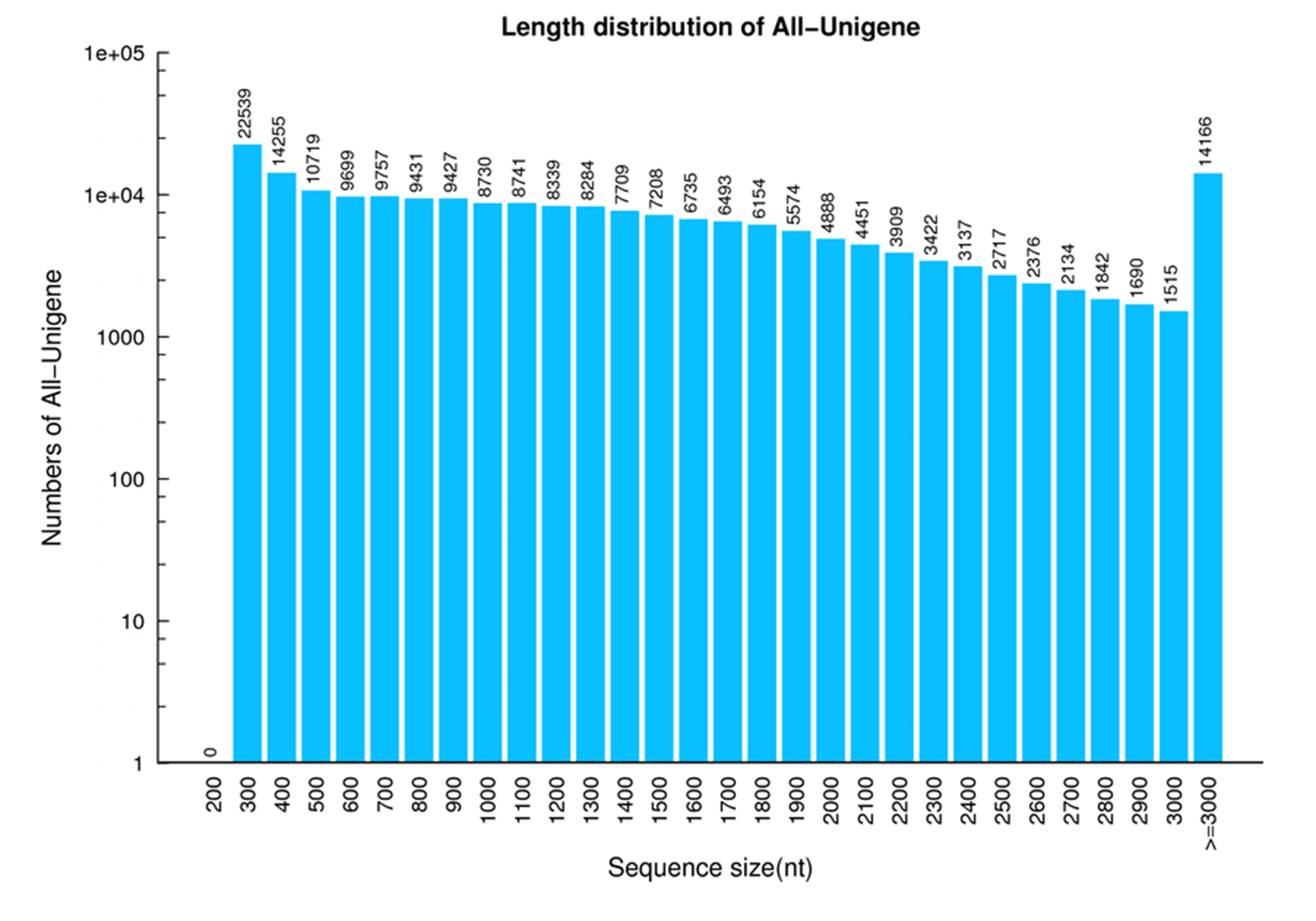

Supplement: Supplementary file 4 — Length distribution of unigenes. (TIFF 5378 kb) [file 12864_2017_4324_MOESM4_ESM.tif]

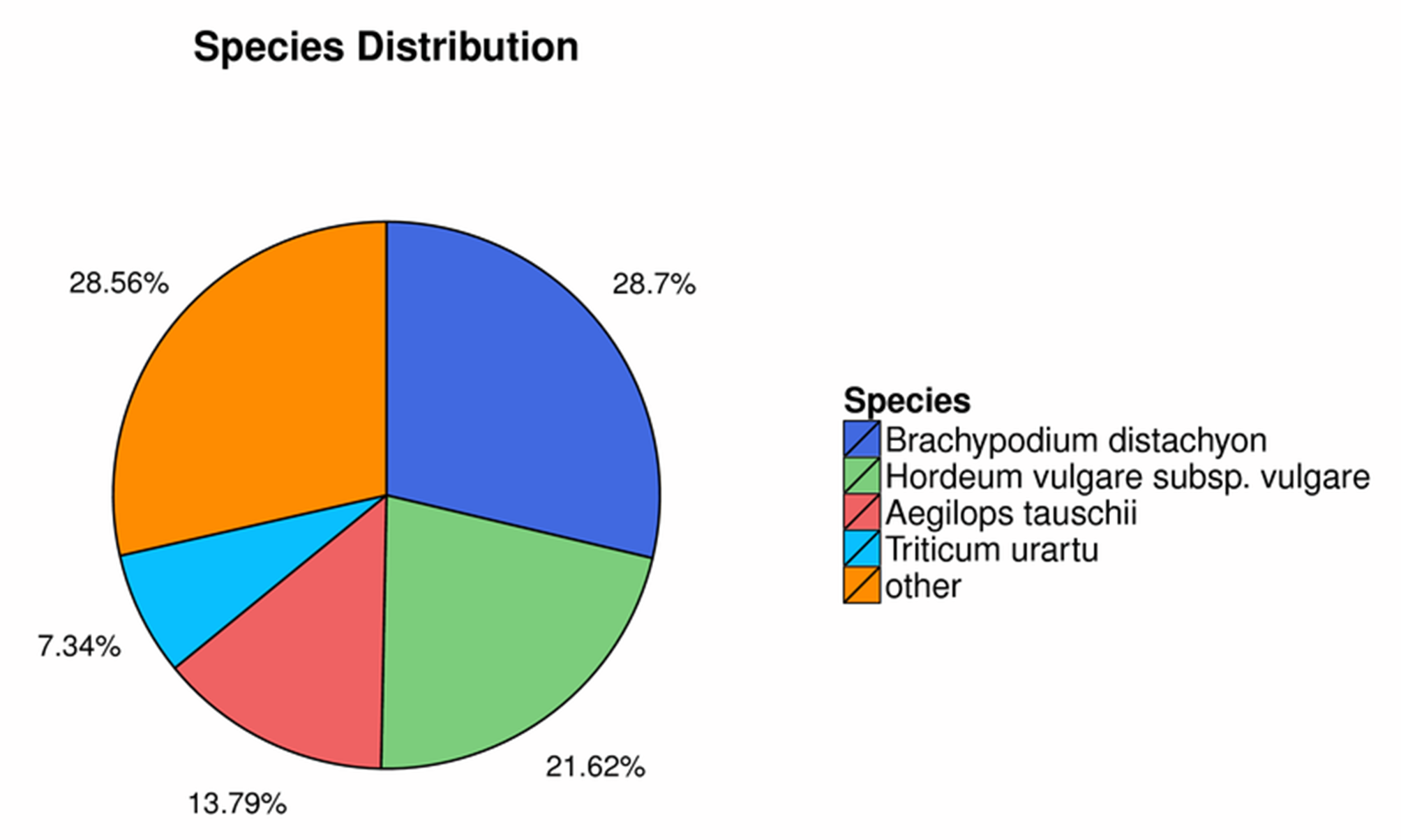

Supplement: Supplementary file 5 — Distribution of annotated species. (TIFF 3199 kb) [file 12864_2017_4324_MOESM5_ESM.tif]

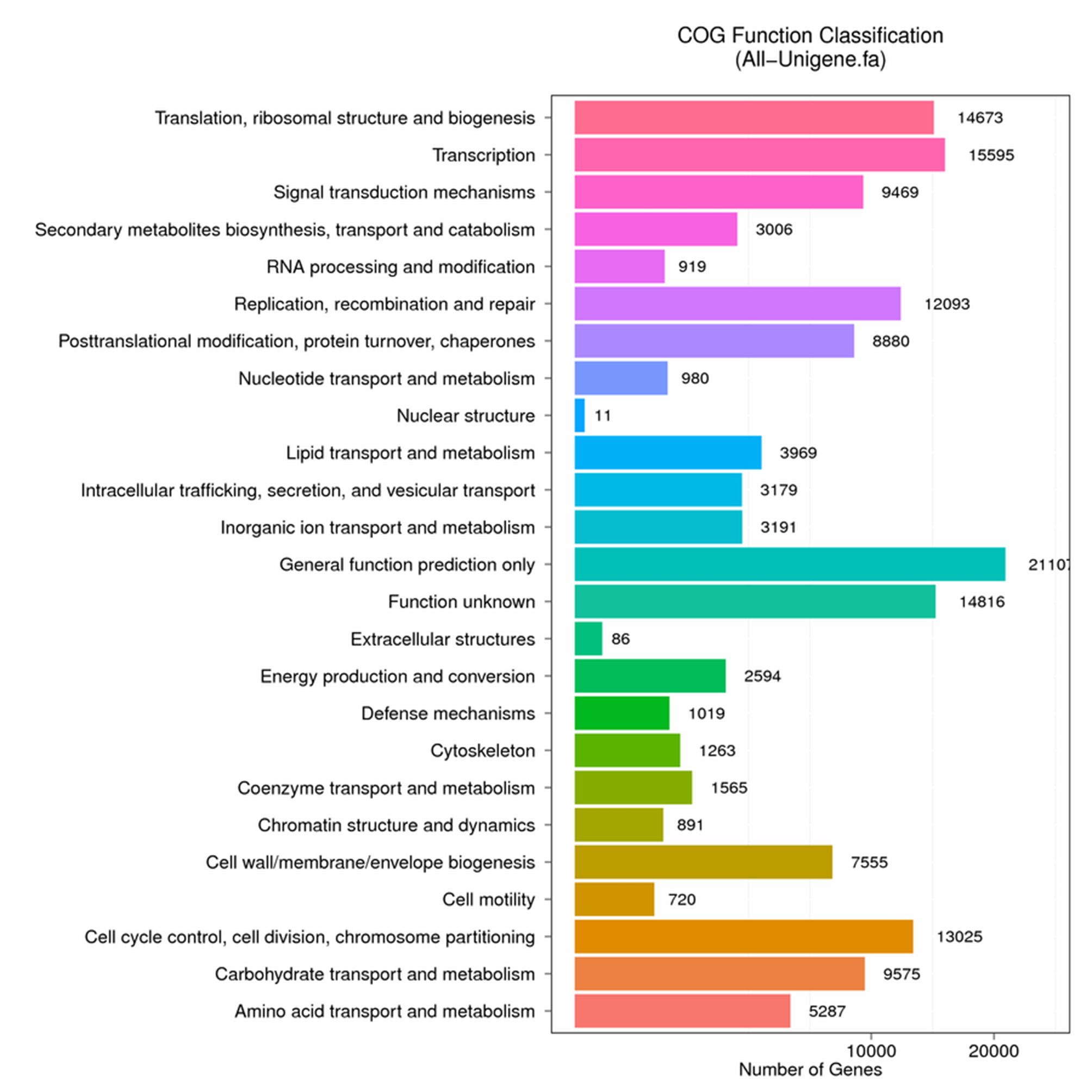

Supplement: Supplementary file 6 — Functional distribution of COG annotation. (TIFF 6509 kb) [file 12864_2017_4324_MOESM6_ESM.tif]
